# The majestic canopy-emergent genus *Dinizia* (Leguminosae: Caesalpinioideae), including a new species endemic to the Brazilian state of Espírito Santo

**DOI:** 10.1007/s12225-017-9720-7

**Published:** 2017-10-06

**Authors:** G. P. Lewis, G. S. Siqueira, H. Banks, A. Bruneau

**Affiliations:** 1 Comparative Plant and Fungal Biology Department, Royal Botanic Gardens, Kew, Richmond, Surrey TW9 3AB UK; 2 Herbário - Reserva Natural Vale, Meio Ambiente, BR 101, km 122, s/n., Caixa Postal 91, Sooretama, Espírito Santo 29.927–000 Brazil; 3 Institut de recherche en biologie végétale and Département de sciences biologiques, Université de Montréal, Montréal, Québec H1X 2B2 Canada

**Keywords:** Fabaceae, fossils, Neotropics, pollen, taxonomy

## Abstract

Since its description, almost 100 years ago, the genus *Dinizia* has been treated as monospecific, comprising the single canopy-emergent species *Dinizia excelsa* Ducke which grows in non-flooded Amazonian forests of Guyana, Suriname and seven states of northern and central-western Brazil. *Dinizia jueirana-facao* G. P. Lewis & G. S. Siqueira, which grows in a restricted area of semi-deciduous Atlantic rain forest in Espírito Santo state, Brazil, is described as a new species in the genus. The new species is also a canopy-emergent of impressive stature. We provide descriptions for both species, a key to species identification, a distribution map and the new species is illustrated. Fossil leaves, inflorescences and fruit provide evidence for a *Dinizia*-like ancestor occurring in south-eastern North America during the Eocene. In contrast to *D. excelsa* where pollen is dispersed in tetrads, the pollen of *D. jueirana-facao* is shed in monads. *D. jueirana-facao* is considered critically endangered following IUCN conservation criteria, whereas *D. excelsa* is assessed to be of least concern. A lectotype is designated for *D. excelsa*.

## Introduction

For almost 100 years the genus *Dinizia* has been treated as monospecific. The genus was first described by Ducke (1922) to accommodate the single species *D. excelsa* Ducke, a rain forest canopy-emergent of impressive stature (some individuals over 60 m tall are recorded from the Brazilian Amazon). Ducke named the tree after his friend José Picanço Diniz, doctor-in-law and philanthropist, thanks to whom botanical exploration in Trombetas was made possible. Burkart (1943) placed *Dinizia* in his tribe Mimozygantheae based on the similar imbricate sepals and indehiscent fruits of *D. excelsa* and *Mimozyganthus carinatus* (Griseb.) Burkart. In addition, both species have a nectary in a distinct hypanthium (Ancibor 1969). The fruits of the two species are, however, vastly different in size and texture and we now know that the two species are not closely related phylogenetically (Luckow *et al.* 2005); the nectary in the hypanthium appears to have evolved independently in the two taxa. The tribe Mimozygantheae has since been disbanded, and *Mimozyganthus* Burkart was shown to belong to tribe Mimoseae, and sister to a clade comprising the two genera *Piptadeniopsis* Burkart and *Prosopidastrum* Burkart (Luckow *et al.* 2005). Luckow *et al.* (2003), based on molecular and morphological data, found *Dinizia* to be more closely related to caesalpinioid genera than to genera in the Mimosoideae. This placement of the genus is supported by it having flowers with a hypanthium, a stylar groove, and imbricate petals, “characters either unusual or unknown among other mimosoids” (Luckow *et al.* 2003). Indeed, Ducke (1949) had already commented on the apparent intermediate position of *Dinizia* between the mimosoids and the caesalpinioids. Barneby *et al.* (2011) excluded *Dinizia* from their treatment of the mimosoids for the *Flora of the Guianas*. Recent molecular studies (Bruneau *et al.* 2008; LPWG 2017) have placed *Dinizia* in the Caesalpinioideae, close to some other members of the *Dimorphandra* group. The genus now belongs to a re-circumscribed Caesalpinioideae, but is not closely related to any genera in the mimosoid clade (LPWG 2017).

## Fossils

Fossil leaves of *Duckeophyllum eocenicum* Herendeen & Dilcher (1990), the co-occurring fossil inflorescence, *Eomimosoidea plumosa* Crepet & Dilcher (1977), and specimens of fossil pods, *Eliasofructus catahoulensis* Herendeen & Dilcher (1990) and *E. claibornensis* Herendeen & Dilcher (1990) may well all represent a single extinct genus, and the vegetative and reproductive fossils have each been compared with the extant species *Dinizia excelsa* (Herendeen & Dilcher 1990). The fossils provide evidence for a *Dinizia*-like ancestor occurring in south-eastern North America during the Eocene. The fossil flower *Eomimosoidea plumosa* has pollen in permanent tetrads, further evidence of an association with *D. excelsa* which also has pollen in tetrads. The fossil fruits are similar to those of *D. excelsa* in several features. Fruit shape and texture, and especially the presence of longitudinal wrinkles near the fruit margin, are all characteristic of the fossils and of *Dinizia excelsa* (Herendeen & Dilcher 1990).

## A new species supported by molecular analyses

Just over a decade ago, Renato Moraes Jesus, then the biodiversity general manager of the Compania Vale do Rio Doce (CVRD) in Linhares, Espirito Santo, Brazil, sent to Kew an unidentified legume specimen (*Folli* 4889) taken from a very large tree growing inside the Reserva Natural Vale. The suggestion, based on field characteristics, that this might be a new species of the mimosoid legume genus *Parkia* R. Br. proved incorrect. Based on inflorescence type, flower morphology and the treeʼs robust woody fruit it seemed more likely that the specimen was related to the caesalpinioid genus *Dimorphandra* Schott. Years later, after the gathering of more field data, together with molecular, palynological, and morphological studies, it is clear that the tree growing in the Atlantic Forest of the Vale Reserve represents a new, second species of the genus *Dinizia*. Molecular phylogenetic studies that have included either or both plastid and nuclear DNA sequences for both *Dinizia excelsa* and *Dinizia* sp. nov. (in particular samples of *Folli* 4884, 4889; e.g., Bruneau *et al.* 2008; Manzanilla & Bruneau 2012; Babineau & Bruneau 2017) find that the two species always group together in a well-supported clade, separate from other clades and species in Caesalpinioideae. Plastid *trn*L-F, *mat*K and *rps*16, and nuclear ITS, *tRALs* and *Leafy* sequences are available for *D. excelsa*, and for the new species, plastid *trn*L-F, *mat*K, *rps*16, and *trn*D-T, and nuclear ITS, *PP1*, *tRALs*, *AIGP*, *EIF3E* and *Leafy* sequences have been analysed (Bruneau *et al.* 2008; Manzanilla & Bruneau 2012; LPWG 2017; Babineau & Bruneau 2017). Although the loci sequenced and the taxon sampling differs amongst studies, and although resolution remains poor overall amongst lineages subtending the mimosoid clade in the Caesalpinioideae, all recent molecular phylogenetic analyses suggest that *Dinizia* occurs in a poorly resolved group that includes *Dimorphandra*, *Campsiandra*, *Mora*, *Burkea* and *Stachyothyrsus*, along with *Tachigali*, *Arapatiella* and *Jacqueshuberia*.

## Pollen

**Fig. 1:**
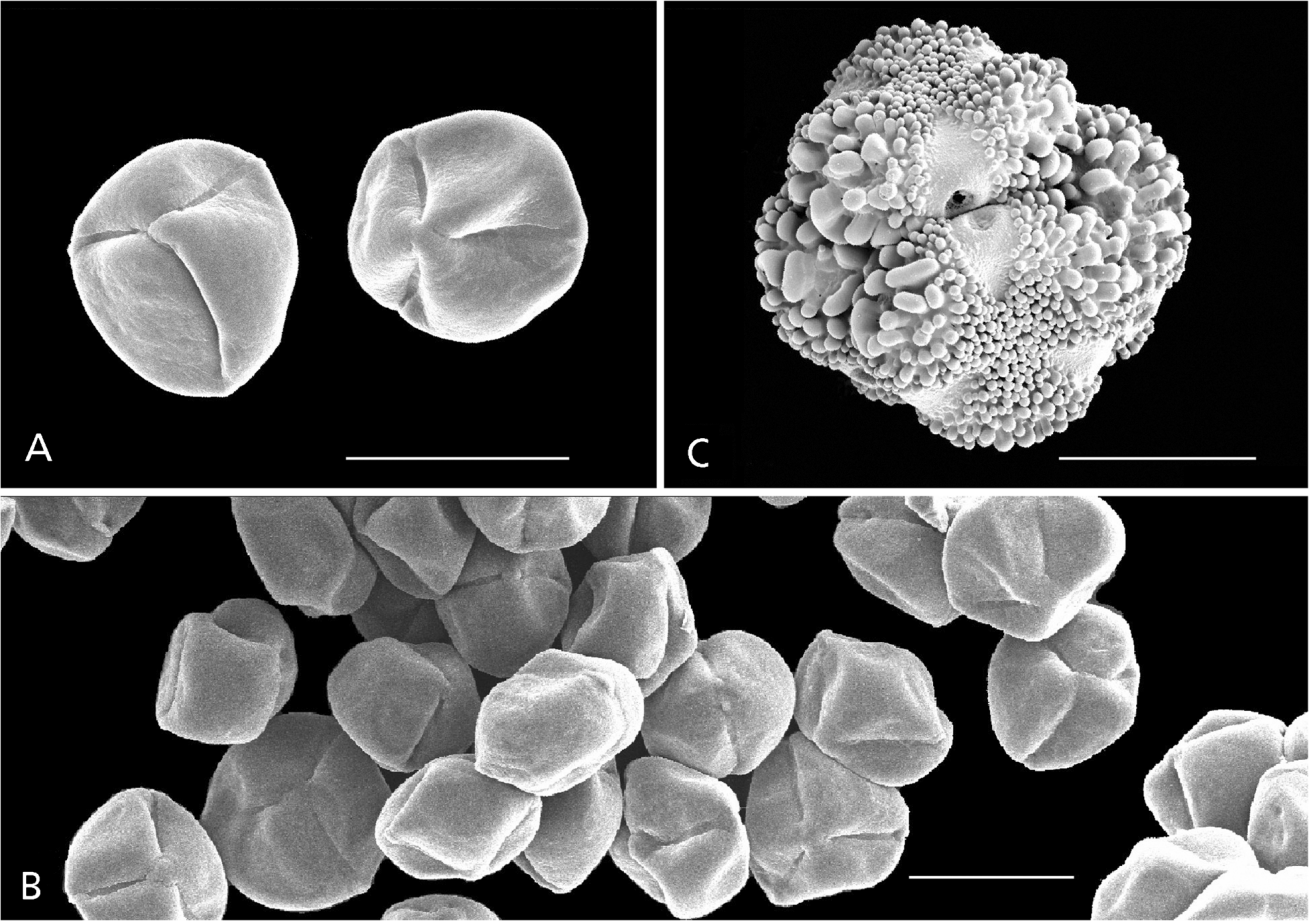
*Dinizia jueirana-facao*. **A** two individual pollen grains; **B** cluster of pollen in monads. **C** *Dinizia excelsa*, pollen a tetrahedral tetrad. Scale bars all 20 μm. sem images: hannah banks.

Guinet (1981), in his seminal contribution to mimosoid legume pollen, stated that, ‘the constant presence of single grains has been found in 14 [mostly species-poor] genera’, while five genera, including *Dinizia* (the genus was then considered to be a member of the Mimosoideae) have either compound or single grains. Thus, fide Guinet (1981), the genus *Dinizia*, then represented by the single species *D. excelsa*, displayed pollen dimorphism, although Guinet published no photographic evidence to support this contention. It is possible that the apparent monads observed by Guinet in *D. excelsa* were the result of acetolysis breaking the tetrads apart. Nevertheless, the new species of *Dinizia* has pollen consistently in monads (Fig. 1A & B) and the genus, as now circumscribed here, therefore evidently displays pollen dimorphism.

*Dinizia* pollen is not the only example of a genus in the legume family that has one species that releases its pollen in tetrads, and another (or others) that releases pollen in monads. Tetrads have arisen at least four times independently in caesalpinioid legumes and are also present in some mimosoid clade legumes. Pollen that is released in tetrads occurs in one or two species of the genera *Bauhinia*, *Diptychandra* and *Afzelia,* with other closely related species being released as normal monads (Sorsa 1969; Ferguson & Banks 1994; Banks 2003; Banks *et al*. 2010). Relatively minor changes in the timing of exine development during ontogeny, and the presence or absence of callose that surrounds microspores during development, can determine whether microspores remain permanently united in calymmate or acalymmate tetrads when mature, or are released as individual monads (Banks *et al.* 2010, Lora *et al.* 2014). The morphology of pollen tetrads varies among the various legume species in which they occur; they are acalymmate in *Afzelia, Bauhinia* and *Dinizia*, and calymmate in *Diptychandra* (Banks & Rudall 2016).

The pollen of *Dinizia excelsa* analysed by us is in tetrahedral tetrads (Fig, 1C) with the individual grains 3-colporate, perforate, gemmate in the polar areas and clavate in the mesocolpial area (i.e they are covered in wart-like lumps, with the lumps more robust in the mesocolpial areas and less so in the polar areas). Guinet (1981) considered *D. excelsa* pollen ornamentation to be unique by the occurrence of well-developed clavae mixed with small verrucae. The tetrads are easily differentiated from the single pollen grains of *Dinizia* sp. nov. (Fig. 1A & B) which have psilate-perforate ornamentation.

## Nitrogen fixation

*Dinizia excelsa* does not nodulate (Moreira *et al.* 1992; Sprent 2001). Branched structures collected from the roots of *Dinizia* sp. nov. (Sergio Faria, pers. comm.) are more likely to be ectomycorrhizal than root nodules housing bacteria (J. Sprent, pers. comm.) and it is thus hypothesised that *Dinizia* sp. nov. also does not nodulate.

## Taxonomic account

Herbarium acronyms follow *Index Herbariorum* (Thiers, continuously updated).

**Dinizia** *Ducke* (1922: 76).

Large forest canopy-emergent *trees*, buttressed or not, bark breaking off in large woody plates. *Leaves* bipinnate, eglandular, the pinnae alternate to subopposite, leaf and pinnae rachises caniculate along upper margin; leaflets alternate, glabrous or the lower surface puberulent to glabrescent. *Inflorescence* a compound raceme; flowers hermaphrodite or functionally male, a nectarial ring at the base of the hypanthium surrounding the centrally placed ovary stipe; petals 5, free, imbricate, attached around the upper rim of the hypanthium; the outer surface of the hypanthium, calyx tube and corolla puberulent, the calyx and corolla lobe margins ciliate; stamens 10, free, glabrous, the filaments inserted around the hypanthium rim, anthers eglandular, dorsally fixed; ovary glabrous to pubescent on its lateral faces, style glabrous, stigma terminal, tubular to slightly funnel-shaped. *Fruit* coriaceous to woody, indehiscent or dehiscent along both sutures, seeds laterally compressed, hard, pleurogram lacking, pollen in monads or tetrads. Type: *Dinizia excelsa* Ducke.

## Key to the species of *Dinizia*

Leaflets in 7 – 14 pairs per pinna, puberulent to glabrescent on their lower surface; individual inflorescence rachis (10 –) 12.5 – 16 cm long, 1 – 1.5 (– 2) cm wide in open flower; buds ellipsoid to obovoid, the calyx completely covering the petals; bracts lanceolate, persistent to caducous; flowers 4 – 5 mm long; fruits coriaceous, indehiscent, red when immature; seeds (10 –) 14 – 15 × 6 – 7 mm; pollen in tetrads; Amazonian rain forest in Brazil and the Guianas……………………………………… **D. excelsa**

Leaflets in (9 –) 15 – 24 pairs per pinna, glabrous on both surfaces; inflorescence rachis 28 – 35 cm long, 3 – 4.5 cm wide in open flower; buds globose, the petals exposed early in development; bracts spathulate, caducous; flowers 8.5 – 10 mm long; fruits woody, dehiscent, yellowish cream or greenish when immature; seeds 25 – 30 × 16 – 19 mm; pollen in monads; Atlantic rain forest……………………**D. jueirana**-**facao**

**Dinizia excelsa** *Ducke* (1922: 76). Type: Brazil, Obidos, Serra do Curumú, 4 Jan. 1914, *Ducke* s.n. (lectotype MG 15304!, designated here), remaining syntypes: *Ducke* s.n. (MG nos. 15774, 15826, 15989, 16177, 17073).

A canopy emergent *tree*, (15 –) 30 – 60 m+, unarmed, trunk cylindrical, bole of larger specimens 15 – 22.5 m, up to 3 m in diam. at soil level, DBH (23 –) 80 cm – 2 m, moderately to strongly buttressed, the buttresses to 4 – 5 m tall (and these “continue off into the forest as raised, laterally compressed roots up to 80 cm high”, *Zarucchi et al.* 2936), crown spreading; bark smooth, white, breaking off in woody plates to reveal a light red-brown or brick-red under bark; heartwood brown to red, without streaks. *Stipules* subulate, 3 – 6 mm long, caducous. *Leaves* bipinnate, eglandular, the petiole terete, 2 – 7.5 cm long, the rachis (4 –) 6 – 28 cm long, caniculate, puberulent; pinnae in 3 – 6 subopposite to strongly alternate pairs, or odd-pinnate with one extra pinna on one side (i.e. total pinnae per leaf 7 to 11 (– 13)), the pinnae 6.5 – 12.5 cm long, the rachis caniculate with raised ridges along each side of the channel, puberulent to pubescent; *leaflets* alternate, in 7 – 14 pairs per pinna, subsessile, oblong, subelliptic, to trapeziform, 12 – 25 × 5 – 11 mm, leaflet apex retuse to rounded, base truncate, inequilateral about the midvein, the lamina much broader on the distal side of the midvein base, the midvein otherwise subcentral to diagonal, secondary venation brochidodromous, but hardly visible, lamina discolorous, the upper surface darker, glabrous (except for a few hairs on the slightly immersed midvein) and nitid, the lower surface sparsely puberulent to glabrescent, including on the prominent midvein, the margins revolute, the pulvinule fleshy, cone-shaped, puberulent. *Inflorescence* a multi-branched, terminal compound raceme, its rachis puberulent; individual racemes over 150-flowered, the peduncle 3 – 15 (– 20) mm long, the rachis (8 –) 12.5 – 16 cm long, the raceme 1 – 1.5 (– 2) cm wide in open flower; *flowers* mostly functionally male (the gynoecium supressed or lacking), fewer flowers in each individual raceme hermaphrodite, all flowers 4 – 5 mm long (from base of pedicel to apex of petals), whitish green to greenish yellow, fragrant, short-pedicellate, the pedicel 0.5 – 1 mm, a persistent to caducous, lanceolate, pubescent, 0.5 mm bract at the base of each flower pedicel, bracteoles lacking, buds globose, the petals exposed early in development; the pedicel, hypanthium, calyx tube and its 5 equal, short, broadly triangular lobes all puberulent with white hairs, the lobe margins ciliate, the hypanthium and calyx tube together 1 – 1.25 mm long; petals 5, free, imbricate, obovate to elliptic, lacking a distinct claw, slightly hooded to dorsally concave, 3 – 4 × 2 – 2.25 mm, sparsely hairy along a central vertical line on the dorsal surface, glabrescent, the margin sparsely to moderately ciliate. *Stamens* 10, white, 3× petal length, the filaments 10 – 12 mm long, very shortly fused at their bases and attached as a ring to the rim of the hypanthium, anthers uniform, dorsifixed, 0.6 – 0.7 mm, anther glands lacking, staminodes lacking. *Gynoecium* red, glabrous, the base short-stipitate, style terminating in a slightly flared (funnel-shaped) hollow stigma. *Fruit* wine-red coloured when fresh (*Simon et al.* 1452), laterally compressed, coriaceous, glabrous, indehiscent, 20.5 – 35 (including a 1.5 – 2 cm stipe) × 4.5 – 8.5 cm, the sutures longitudinally wrinkled and appearing almost winged, the upper “wing” ± 1 cm wide, 7 – 12-seeded. *Seeds* oblong to elliptic, sometimes slightly narrower in the middle, (10 –)14 – 15 × 6 – 7 mm, laterally compressed, black, hard (the texture of a pebble), the surfaces with a network of minute fracture lines, pleurogram absent, the apex narrowing to a terminal funicle attachment, < 1 mm. *Pollen* in acalymmate tetrahedral tetrads with the individual grains 3-colporate, and ornamentation gemmate in the polar areas and clavate in mesocolpial areas (Fig. 1C). Root nodules lacking.

**Map 1:**
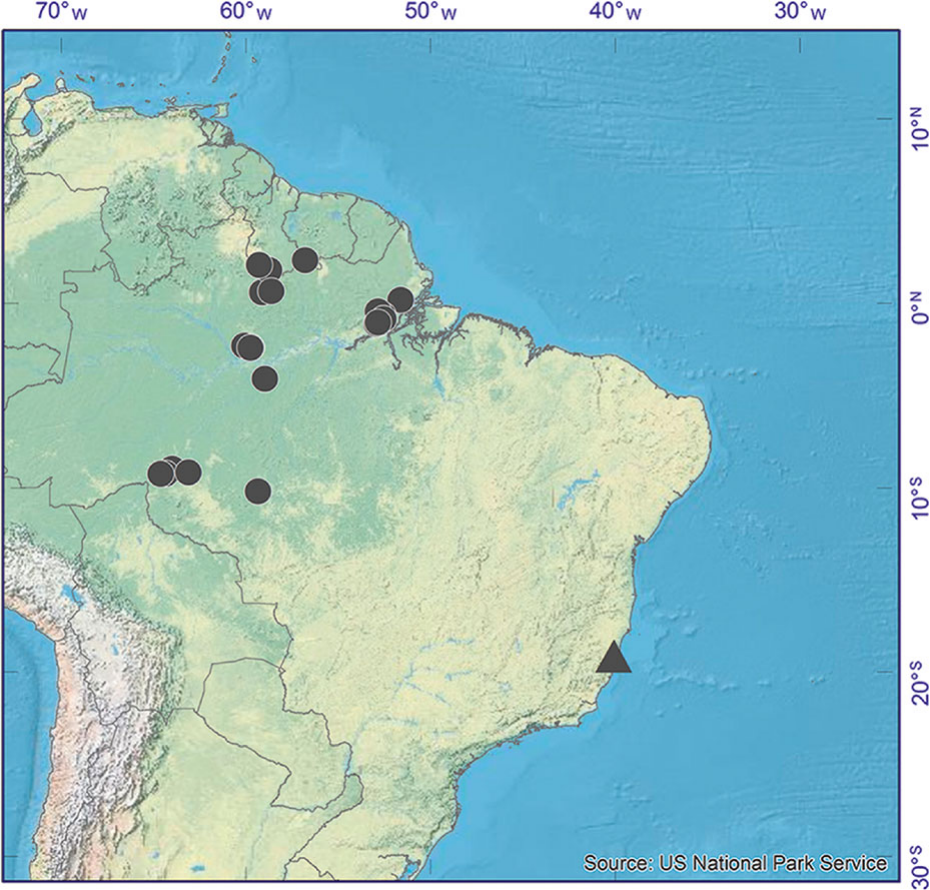
Distribution of *Dinizia excelsa* (black circles) and *D. jueirana-facao* (black triangle) in Brazil and the Guianas.

**distribution**. Guyana, Suriname and Amazonian Brazil (in the northern and central-western states of Amapá, Amazonas, Mato Grosso, Pará, Rondônia, Roraima and Tocantins). Also recorded from the state of Acre by Lorenzi (1992). Map 1.

**specimens examined**. **brazil**: **Amapá**, Serra do Navio, Rio Amapari, trail to Rio Araguary, 2 km from camp, 6 Nov. 1954 (fr.), *Cowan* 38124 (K!); Mun. de Mazagão, Camaipi, 0°10'N, 51°37'W, 23 Dec. 1984 (fr.), *Mori et al.* 17511 (K!, NY); Camaipi, c. 0°10'N, 51°37'W, 17 Sept. 1983 (fr.), *Mori et al.* 16236 (K!, NY); 19 Sept. 1983 (st.), *Mori et al.* 16385 (K!, NY); 19 Sept. 1983 (st.), *Mori et al.* 16401 (K!, NY); 19 Sept. 1983 (st.), *Mori et al.* 16408 (K!, NY); **Amazonas**, Mun. de Axinim, basin of Rio Abacaxis, lower Rio Paca, 4°07'S, 58°58'W, 1 July 1983 (fr.), *Zarucchi et al.* 2936 (INPA, K!, NY); Mun. de Manaus, c. 90 km N de Manaus, 02°19'S, 60°05'W, 19 Aug. 1995 (fl.), *Nee & Dick* 46239 (K!, NY); Manaus, 8 Aug. 1942 (fl.), *Ducke* 975 (K!); Manaus, Colonia Campos Salles, 27 July 1932 (fl. & fr.), *Ducke* 24201 (K, 2 sheets!, RB); Manaus, experimental station, km 60, 17 July 1977 (seed only), *da Silva* 295 (K!); Rio dos Pombos (a tributary of the Yuma river), 74 km E of the Aripuanã river, 21 June 1979 (fl.), *Calderón et al.* 2646 (INPA, K!); Distrito Agropecuário, 2°24'26" – 2°25'31"S, 59°43'40" – 59°45'50"W, 7 July 1990 (fl.), *Mori et al.* 21327 (K!, NY); **Mato Grosso**, Rio Aripuanã, road from Nucleo Pioneiro de Humboldt to Rio Juruena, km 8, 10°12'S, 59°21'W, 25 Oct. 1973 (fr.), *Berg & Steward* P19869 (K!, NY); **Pará**, near the Rio Jaburuzinho, 12 July 1923 (buds & fr.), *Ducke* s.n. (K!, RB No. 16810); Gurupá, 16 May 1916 (buds), *Ducke* s.n. (RB No. 10240, 2 sheets, barcodes 00539872! and 00547527!, MG No. 16177!); Gurupá, 25 Jan. 1916 (fr.), *Ducke* s.n. (MG 15982, two sheets!); Rio Tapajoz, região das cachoeiras inferiors (Poção), 26 June 1918 (buds, fr.), *Ducke* s.n. (RB No. 10241, barcode 00539873!, MG No. 17073); Rio Tapajoz, Bella Vista, 6 Dec. 1915 (fr. & seeds), *Ducke* s.n. (RB No. 10239, barcode 00539874!, MG No. 15826); Obidos, Serra do Curumú, 1 Oct. 1915 (fr.), *Ducke* s.n. (MG 15774!); Obidos, Serra do Curumú, 4 Jan. 1914 (fr.), *Ducke* s.n. (lectotype: MG 15304!); Mt Dourado, Água Azul, 1°7'S, 52°55'W, 4 Jan. 1988 (fr.), *Pires & Silva* 1907 (K!); Mun. Almeirim, Mt Dourado, 6 July 1987 (fl.), *Pires et al.* 1713 (K!); 0°40'S, 52°35'W, 20 Jan. 1988 (fr.), *Pires & Silva* 1955 (K!); 0°47'S, 52°42'W, 31 May 1988 (fr.), *Pires & Silva* 2172 (K!); Estação Ecol. Jarí, 0°27'S, 52°51'W, 6 Jan. 1988 (fr.), *Pires & Silva* 1916 (K!); Monte Dourado, 1°03'S, 52°51'W, 8 June 1988 (fr.), *Pires & Silva* 2214 (K!); Monte Dourado, 00°52'S, 52°33'W, 14 June 1988 (st.), *Pires & Silva* 2222 (K!); 1°03'S, 52°51'W, 14 July 1988 (buds), *Pires* 2305 (K!); **Rondônia**, Porto Velho, ao longo da BR-364, 61 km Leste de Jaci Paraná, ramal 500 m ao Sul, 08°58'17"S, 63°59'16"W, 12 April 2012 (fr.), *Simon et al.* 1452 (CEN, K 2 sheets!); 09°14'39"S, 64°20'56"W, 14 April 2012 (buds), *Simon et al.* 1481 (CEN, K!); 09°15'47"S, 64°37'02"W, 15 Aug. 2010 (fr.), *Pereira-Silva et al.* 15634 (CEN, K!); Mun. de Santa Barbara, rodovia BR-364, km 120, 9°10'S, 63°07'W, 29 May 1982 (fl.), *Teixeira et al.* 871 (INPA, K!); **Roraima**, Mun. São João de Baliza, Rio Jatapuzinho, 0°35'N, 59°07'W, Nov. 1994 (fr.), *Milliken* 2258 (K!). **guyana**: U.Takutu-U., Essequibo Region, Kamoa Mts, 1°47'22"N, 58°44'18"W, 25 May 1997 (fr.), *Clarke* 4956 (K!, US); Gunn’s, Essequibo R., 30 Sept. 1989 (fl.), *Jansen-Jacobs et al.* 1900 (K!, U); Essequibo, Kuyuwini R., 0 – 2 km NW of camp, 02°04'N, 59°17'W, 18 July 1996 (fr.), *Clarke* 2257 (K!, US); Simuni Creek, Rupununi R., a few miles N of Kanaku Mts, 8 August 1931 (fl.), *Davis in Forest Department of British Guiana* field no. D128, record no. 2119 (K, 3 sheets!). **suriname**: Sipaliwini, 3 km S (190°) from Kwamalasamutu village centre, 2°19'30"N, 56°47'20"W, 22 Feb. 2006 (fr.), *Hoffman* 6691 (K!, US).

**habitat**. The species clearly prefers non-flooded environments and is recorded from non-inundated moist forest, non-flooded upland mixed forest, “floresta ombrofila mista”, tropical forest on terra firme, tropical upland evergreen forest and tropical dry forest, at elevations from 50 – 490 m.

**conservation status**. *Dinizia excelsa* is geographically widely dispersed in seven Brazilian states, Guyana and Suriname and has a tendency to be gregarious (*Forest Dept. of British Guiana* field no. D128, record no. 2119; and da Silva *et al.* 1977). The estimated extent of occurrence (EOO) exceeds the thresholds for a threatened category according to IUCN criteria version 3.1 (IUCN 2012) and it is suspected that the area of occupancy (AOO) also exceeds these thresholds. It is therefore assessed as being of Least Concern, although it is not known how frequently encountered the tree is today across its distribution range, and it is evident from the literature that its wood has been widely used (see under notes). The species is not protected under CITES regulations.

**phenology**. Collected in flower in Brazil from April to August, and in fruit throughout the year (no fruiting collections seen from February or March); in Brazil the main flowering period in most states is July and August; collected in flower in Guyana in August and September and in fruit in May and July, and in Suriname a single fruiting specimen (in K) was collected in February.

**common names**. “Angelim”, “angelim pedra”, “angelim vermelho”, “paricá” (Brazil); “Awaraimë” (Trio, Suriname); “parakwa” (Wapisiana, Guyana). Lorenzi (1992: 176) also includes the popular names: “angelim falso”, “faveira”, “faveira-dura”, faveira-ferro” and “faveiro-do-grande”.

**notes**. The species is notable for the hardness of its wood and its bark breaking off in woody plates which accumulate in piles at the base of the tree. Usually the most terminal raceme in the compound inflorescence flowers first, then the basal racemes open, followed by those above; the flowers in each raceme open somewhat irregularly, but generally from the base to the apex of the raceme; freshly opened flowers are strongly fragrant (*Nee & Dick* 46239). *Dinizia excelsa* is reported to be pollinated by bees (Ribeiro *et al.* 1999). The wood is very resistant and difficult to work, but has been widely used for railway sleepers, in civil and naval construction, cabinetwork and joinery (da Silva *et al.* 1977), and for battens, props, beams, girders, posts, stakes, door and window frames, floor boards, carts, wagons and bridges (Lorenzi 1992). The wood density of *D. excelsa* is recorded as between 0.83 – 0.91 g/cm^3^ by Fearnside (1997) and as 0.9 – 1.2 g/cm^3^ by Richter & Dallwitz (online version 2009), who also describe the wood odour as distinct, very unpleasant and persistent.

In the protologue of *Dinizia excelsa*, Ducke (1922) cited six specimens that he had collected in Pará between 1914 and 1918 (MG herbarium numbers: 15304, 15774, 15826, 15989, 16177 and 17073) without choosing a holotype. The six collections should thus be considered as syntypes; all are still housed in the Museu Goeldi (MG) herbarium, with some duplicated in the herbarium of the Rio de Janeiro Botanic Gardens (RB). A number of the specimens in MG carry original field labels in Ducke’s handwriting, but no one specimen bears both flower and fruit material. The specimen in the best condition, which fits the description of foliage and fruits presented in the original description, and which includes an original label in Ducke’s hand, is MG15304 from the Serra do Curumú collected on the 4^th^ of January 1914. This specimen is thus designated as the lectotype of *D. excelsa*. The other specimens cited in the speciesʼ protologue become remaining syntypes.

**Dinizia jueirana**-**facao** *G. P. Lewis & G. S. Siqueira* **sp. nov.** Type: Brazil, Espírito Santo, Linhares, Reserva Natural Vale, 30 July 2004 (fl.), *D. A. Folli* 4889 (holotype CVRD!; isotypes HUEFS!, K!).

http://www.ipni.org/urn:lsid:ipni.org:names:60475109-2

**Fig. 2:**
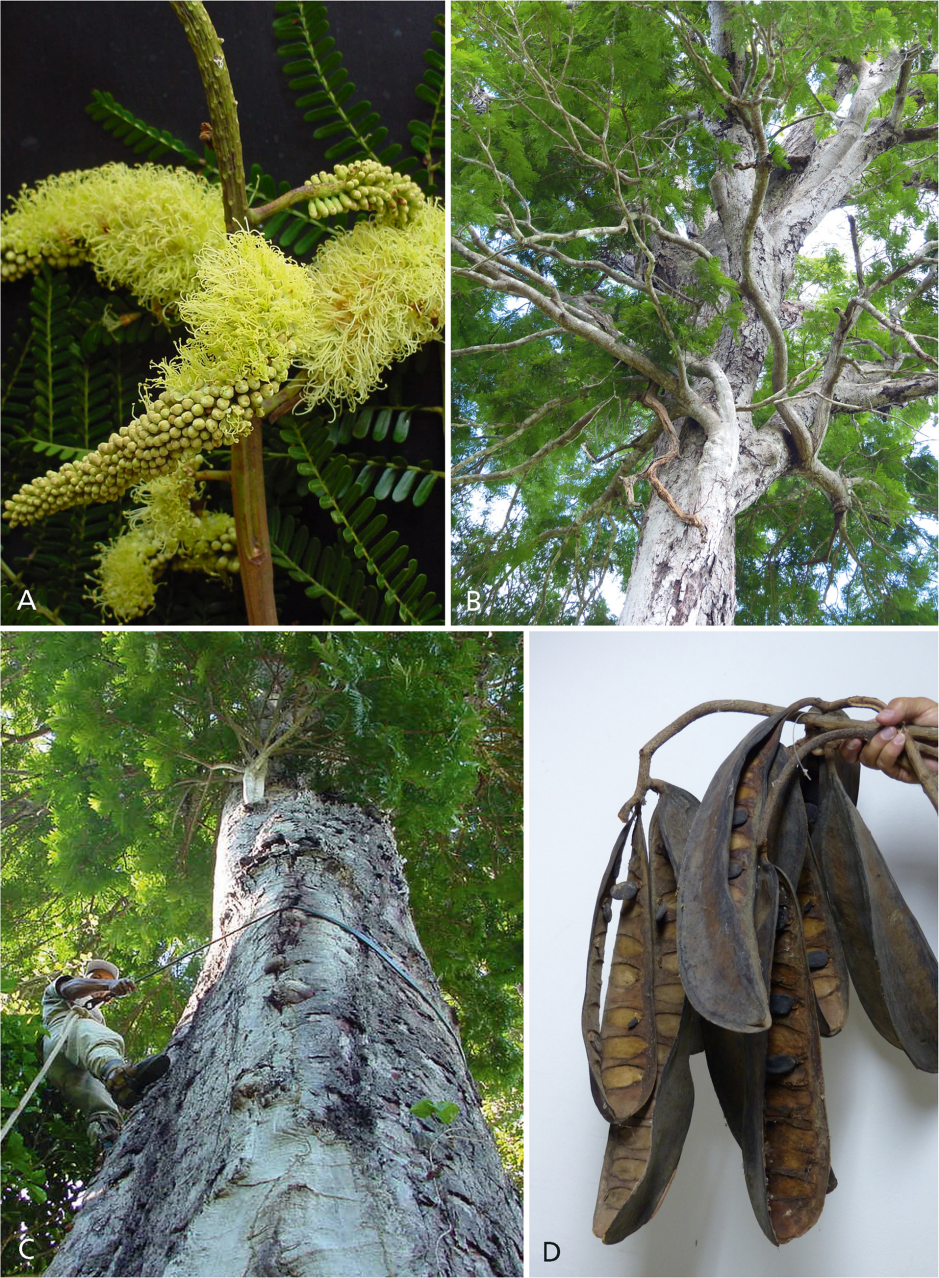
*Dinizia jueirana-facao*. **A** part of a compound inflorescence; **B** part of crown of type specimen; **C** trunk of type specimen; **D** dehisced fruits. photos: **a**, **c** domingos a. folli, **b** **,** **d** g. p. lewis.

**Fig. 3:**
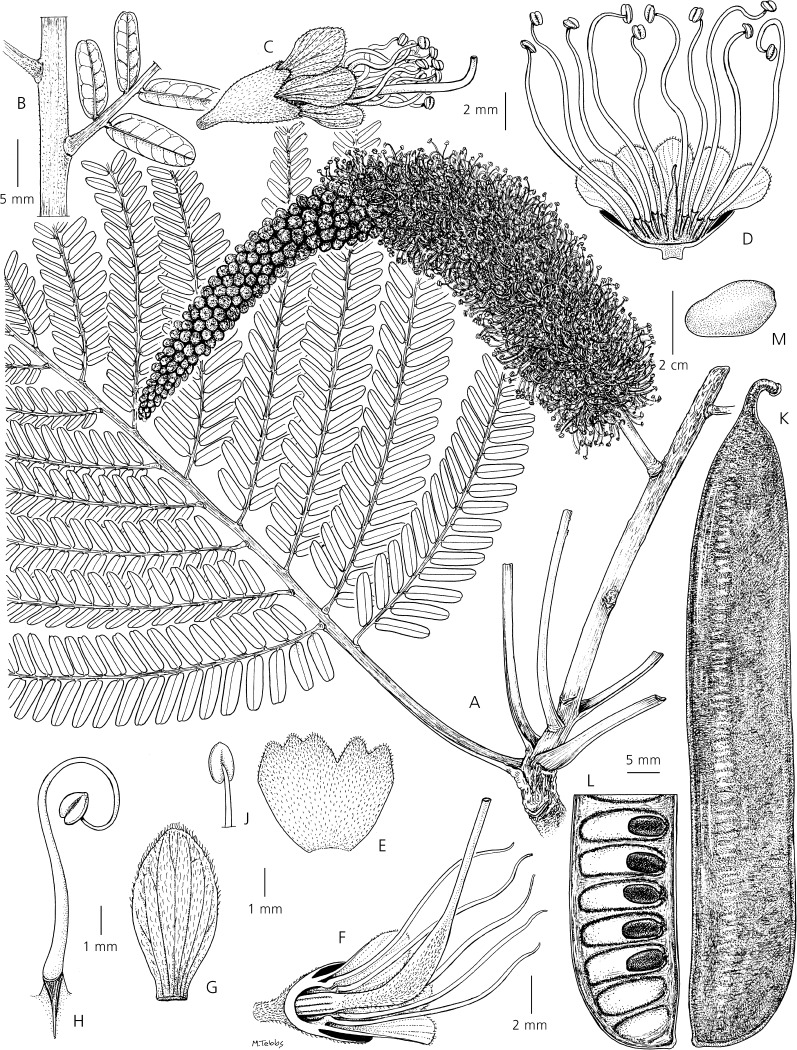
*Dinizia jueirana-facao*. **A** flowering branch and part of a bipinnate leaf; **B** leaflets at the base of a single pinna; **C** hermaphrodite flower; **D** functionally male flower opened to show stamen filaments and suppressed gynoecium development; **E** calyx opened out, outer surface; **F** longitudinal section of hermaphrodite flower to show gynoecium; **G** petal, outer surface; **H** stamen; **J** anther; **K** fruit; **L** part of a single valve of dehisced fruit with seeds attached; **M** seed. **A – J** from *Folli* 4889 (K), **K – M** from *Folli* 4484 (K). drawn by margaret tebbs.

**Fig. 4:**
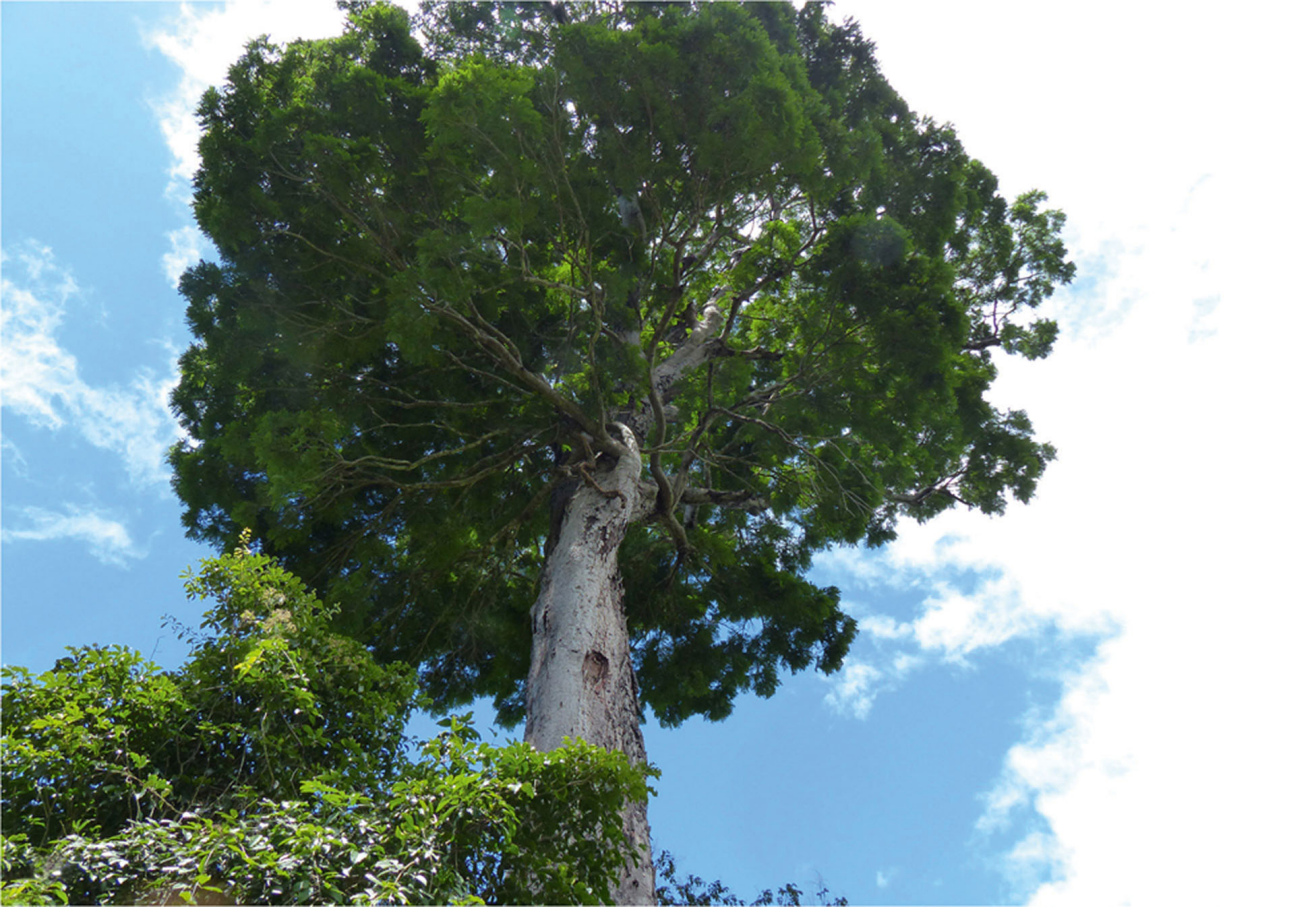
*Dinizia jueirana-facao,* type specimen. photo: g. p. lewis.

*Tree*, 19 – 40 m, unarmed, trunk 10 – 22 m before first major branching, DBH up to 1.56 m, circumference at breast height (1.40 –) 1.90 – 4.90 m, diam. of crown 10 – 20 m, bark grey, frequently breaking off in large woody plates, sap clear and watery. *Stipules* not seen. *Leaves* (including measurements from a 10 year old, 6 m tall, planted tree), alternate to spirally arranged, bipinnate, (25.5 –) 35 – 96 cm long (including the petiole), eglandular; petiole 5.5 – 10 cm long, flattened on upper edge near its base, angular at the margins, ± rounded along lower edge, leaf rachis caniculate along upper margin, the channel becoming more pronounced towards the distal end; pinnae in (9 –) 15 – 19 alternate to sub-opposite pairs per leaf (sometimes an extra terminal pinna on one side of the rachis, the total number of pinnae per leaf thus either even or odd), 9.5 – 15.5 cm long, the proximal and distal pinnae shorter and with fewer leaflet pairs than the median pinnae, the pinna rachis caniculate along its upper edge with pronounced raised puberulent ridges on either side of the channel; *leaflets* alternate to subopposite, sessile, (9 –) 15 – 23 (– 24) pairs per pinna, their blades sub-rhombiform, coriaceous, 8 – 23 × 2.5 – 6 mm, glabrous on both surfaces, somewhat discolorous on drying, the upper surface darker and shiny, the apex rounded to very shallowly retuse, the base subtruncate to rounded, inequilateral with the distal side of the blade distinctly broader than the proximal side, blade margin entire, slightly thickened, slightly revolute, the midvein ± central, slightly prominent to slightly immersed on the upper surface, distinctly prominent on the lower surface, a fleshy cone-shaped pulvinus at the base of the midvein, secondary venation not visible on the upper surface, brochidodromous but obscure on the lower surface. *Inflorescence* an erect, terminal, large woody compound raceme, exserted from the surrounding foliage, the racemes in groups of two or more subtended by a woody, 15 – 30 cm long, densely tomentose to puberulent, rust-coloured, primary woody peduncle (the colour contrasting with the white indumentum of the leaf petioles), the individual inflorescence rachis sparsely puberulent, 28 – 35 cm long, 3 – 4.5 cm wide (in open flower), longitudinally ridged (when dry), each raceme with hundreds of pedicellate flowers (fallen flowers leaving narrowly ellipsoid sunken pedicel scars on the rachis, these occasionally filled with a small resin droplet), *flowers* bright yellow, hermaphrodite (although some apical flowers appearing functionally male due to suppression of gynoecium development), 8.5 – 10 mm long (from base of the robust 1.5 – 2 mm pedicel to the tips of the petals), a caducous, spathulate, stipitate, puberulent, 1.5 – 2 mm bract inserted on an inflorescence ridge directly below each flower pedicel, but these obscure and most evident below buds; calyx valvate in bud, the buds ellipsoid to obovoid, the broadly acute calyx lobes spreading apart in a symmetrical star shape to reveal the petals beneath, calyx of mature flowers campanulate, coriaceous, puberulent on the tube and 5 lobes, the minute white hairs especially dense on the lobe apices, the tubular hypanthium 2 – 2.5 mm long, a darkened nectarial zone at its inner base, the calyx tube c. 2 mm long, the subequal lobes 1.25 – 1.5 mm; petals free, imbricate, 5.5 – 7 × 3 – 3.5 mm, subequal (the median petal the smallest), inserted around the upper margin of the hypanthium, the slightly reflexed blade glabrous on its inner surface, moderately pubescent with white hairs over most of the outer surface, the petal margins densely ciliate, the broad claw almost as wide as the blade; stamens 10, free, c. 20 – 25 mm long, inserted in two whorls (one slightly higher than the other) along the upper margin of the hypanthium, glabrous, anthers uniform, dorsifixed, anther apex with a short thickened connective, anther glands lacking, staminodes lacking; ovary c. 9 mm long, short-stipitate, the stipe 3 – 4 mm, inserted centrally within the hypanthium, pubescent, especially on the two lateral faces, the style glabrous and tapering to an apical, tubular, glabrous stigma. *Fruits* scimitar-shaped, ± falcate, woody, yellowish cream to greenish when immature, maturing dark brown to black, 40 – 46 × 8.5 – 10 cm, smooth, glabrous, the upper and lower sutures thickened and longitudinally ridged, dehiscent along both sutures, the woody exocarp raised between the seed chambers, 13 – 15-seeded. *Seeds* black, hard (the texture of pebbles), elliptic to obovate in outline, 25 – 30 × 16 – 19 mm, laterally compressed, sub-nitid along the margins, pleurogram lacking, the lateral surfaces minutely pitted and with a fine network of fracture lines (only visible with a ×10 lens), the apical funicular attachment point 2 – 4 mm long. *Pollen* oblate, tricolporate, with psilate aperture membranes, c. 30 μm in diam., with psilate-microperforate ornamentation, the mesocolpial areas smooth, the areas around the aperture margins more rugulate and with a higher density of perforations, the aperture margins project over the endoaperture areas, the apices of the apertures fork into indentations that almost join around the apocolpium to form a weakly syncolporate pattern. *Root nodules* lacking. Figs 1A & B, 2, 3 & 4.

**recognition**. *Dinizia jueirana-facao* differs from its sister species *D. excelsa* in having leaflets in (9 –) 15 – 23 (– 24) pairs per pinna (vs 7 – 14 pairs), the leaflets completely glabrous (vs puberulent to glabrescent on their lower surface), its individual racemes 28 – 35 × 3 – 4.5 cm (vs 10 – 18 × 1 – 2 cm), buds ellipsoid to obovoid (vs globose), flowers 8.5 – 10 mm long (vs 4 – 5 mm long), its floral bracts spathulate and caducous (vs lanceolate and often persistent), its fruit woody and dehiscent along both sutures (vs indehiscent), seeds 25 – 30 × 16 – 19 mm (vs (10 –) 14 – 15 × 6 – 7 mm); and pollen in monads (vs tetrads).

**distribution**. *Dinizia jueirana-facao* is currently known only from two locations, one (19°08'52.0"S, 40°05'16.4"W) in the Reserva Natural Vale in Linhares, northern Espirito Santo state, Brazil, and the second (19°05'12.1"S, 40°10'41.2"W) just outside the reserve in the surroundings of the small hamlet of Santa Luzia Sooretama. Map 1.

**specimens examined**. **brazil**: Espírito Santo: Linhares, Reserva Natural Vale, 20 March 2003 (fr.), *Folli* 4484 (CVRD!, HUEFS!, K!); 30 July 2004 (fl.), *Folli* 4888 (CVRD 8816!, HUEFS!); 30 July 2004 (fl.), *Folli* 4889 (holotype CVRD 8814!, isotypes HUEFS!, K!); Sooretama, UTM 37606, 7889162, 8 Oct. 2014 (fl.), *Folli* 7270 (CVRD 15119!, RB!); Sooretama, 28 Sept. 2015 (fr.), *Folli* 7409 (CVRD 15506!); Reserva Natural Vale, 19°08'50"S, 40°05'12"W, 21 May 2013 (st., 6 m sapling tree), *Neves et al.* 1220 (RB 574861) (BHCB, E!, HUEFS, K!).

**habitat**. An emergent tree in semi-deciduous forest and mata ciliar in the Reserva Natural Vale, an area of 22,000 hectares of pristine Atlantic Forest. This is the largest protected area of semi-deciduous forest in eastern Brazil. Also known from mata de tabuleiro, in the surroundings of Sooretama, just outside the Vale Reserve. Growing at elevations of 40 – 150 m above sea level.

**conservation status**. There are two localities of *Dinizia jueirana-facao*, one within the Reserva Natural Vale and one just outside it, in the surroundings of a small settlement known as Santa Luzia Sooretama, Espirito Santo. In the Reserve only 12 adult trees are known, these distributed across an area of 42.99 hectares (UTM: 385596, 7882465). The locality outside the Reserve also has between ten and 12 trees, these dispersed over an area of 64.81 hectares (UTM: 376061, 7889162). To date, the species is only known from these two small areas, which together contain less than 25 adult trees. The species, especially outside the Reserva Natural Vale, is threatened by habitat loss as a consequence of deforestation due to urban development, agriculture, livestock farming and mining. Although one of the localities is inside a protected area, this is owned by the private mining company Vale and if the company was ever to fall on hard times the reserve could lose its protection. The species is assessed as Critically Endangered (C2a(i,ii)+D) according to IUCN criteria version 3.1 (IUCN 2012), due to its very small and restricted population, combined with an inferred continuing decline in the number of mature individuals based on its habitat deforestation rates.

**phenology**. Flowering and fruiting times are poorly known and considered to be unpredictable. Collected in flower in July and October and in fruit in March, July and September. It is assumed that the large woody fruits take many months to reach full maturity.

**etymology**. The species name is taken directly from the local name, “jueirana-facão”, for the tree in Espirito Santo. In the Reserva Natural Vale, the large legume tree *Parkia pendula* (Willd.) Benth ex Walp. is known as jueirana-vermelha and the new *Dinizia* species, which has a very similar bark which breaks off in large woody plates, but much larger fruits, is locally differentiated by replacing vermelha (Portuguese for red) with facão (Portuguese for large knife or machete), because the woody fruits of *D. jueirana-facao* have the appearance of a machete sheath or scabbard. According to the International Code of Nomenclature for algae, fungi and plants (McNeill *et al.* 2012) an epithet can be a word in apposition (Art. 23.1) and taken from any source whatsoever (Art. 23.2), but the Code does not give clear guidance on diacritical signs, just ruling (Art. 60.6) that “the [diacritical] signs are to be suppressed with the necessary transcription of the letters so modified” but without elaborating on what “necessary transcription” means beyond the cited examples, which do not include ã. We thus transcribe the ã as a in the specific epithet here chosen for the new species.

Jueirana is thought to be derived from the Tupi word yuá-rana. Yuá (or Juá) is a Tupi common name for several different plant species, especially those in the Solanaceae with round, spiny fruits (Andrade 2006; Sampaio 1987). Rana in Tupi means similar to, so yuá-rana or jueirana means false juá (or similar to juá), although there is little resemblance between the new legume species and any Solanaceae. A number of place names in Brazil are derived from jueirana or an orthographic variant of this.

**notes** **.** *Dinizia jueirana-facao*, as currently known, is a narrowly restricted species endemic to a small area of Atlantic forest in the Brazilian state of Espirito Santo. Although a tree of shorter stature, and lacking buttresses, many of its vegetative and reproductive morphological characteristics are greater in number and/or size than those seen in its widespread Amazonian sister species, *D. excelsa*. *D. jueirana-facao* has leaflets in (9 –) 15 – 23 (– 24) pairs per pinna (7 – 14 pairs per pinna in *D. excelsa*), the leaflets glabrous (vs puberulent to glabrescent on their lower surface), its individual racemes 28 – 35 × 3 – 4.5 cm (vs 10 – 18 × 1 – 2 cm) in open flower, its flower buds ellipsoid to obovoid (vs globose), its flowers 8.5 – 10 mm long (vs 4 – 5 mm long), its floral bracts spathulate and caducous (vs lanceolate and often persistent), its fruit woody and dehiscent along both sutures (vs indehiscent), its seeds 25 – 30 × 16 – 19 mm (vs (10 –) 14 – 15 × 6 – 7 mm), and its pollen in monads (vs tetrads). *D. jueirana-facao* is critically endangered and presently known from less than 25 trees in two small areas, of which only one locality is inside a protected reserve. The type collection of the new species is from one of the largest trees growing inside the reserve.
